# Expanding codon size

**DOI:** 10.7554/eLife.78869

**Published:** 2022-05-11

**Authors:** Tarana Siddika, Ilka U Heinemann, Patrick O’Donoghue

**Affiliations:** 1 Department of Biochemistry, The University of Western Ontario London Canada; 2 Department of Biochemistry, Department of Chemistry, The University of Western Ontario London Canada

**Keywords:** genetic code expansion, tRNA, directed evolution, four base codons, DNA, bases, *E. coli*

## Abstract

Engineering transfer RNAs to read codons consisting of four bases requires changes in tRNA that go beyond the anticodon sequence.

**Related research article** DeBenedictis EA, Söll D, Esvelt KM. 2022. Measuring the tolerance of the genetic code to altered codon size. *eLife* **11**:e76941. doi: 10.7554/eLife.76941.

Cells use a genetic code to translate the information contained in DNA and RNA sequences into the amino-acid building blocks that make up a protein (Nirenberg et al., 1965; Söll et al., 1965). DNA molecules are chain-like structures consisting of two entwined strands that encode information using an ‘alphabet’ of four nucleotide building blocks (made up of a sugar and a phosphate group, and one of four nitrogenous bases, A, C, G and T).

Some segments of the DNA genome are instructions for making proteins, and a messenger RNA molecule (mRNA) is produced from the DNA template for each protein-coding gene. On the ribosome, molecules known as transfer RNAs (tRNAs) decode the information in mRNAs by reading it in three-letter groups, also known as codons, allowing for 64 unique triplet combinations (three of which are used as stop signals). Despite this wealth of codons, most organisms usually use just 20 amino acids, making the code redundant as several codons can code for the same amino acid.

Expanding or modifying the genetic code may enable scientists to use cells as factories for making an array of molecules, which could further therapies based on proteins, and in even grander schemes, to enable the creation of artificial life forms. Recent engineering efforts have successfully produced proteins using 22 or even 23 different amino acids, rather than the usual 20 (Wright et al., 2018; Tharp et al., 2021). However, the standard triplet codons cannot be easily reassigned into new amino acids, because even though decoding the 64 codons allows for redundancy, all codons are assigned to a specific amino acid in an organism. Reassigning a codon to a new amino acid would drastically change the organisms’ protein composition.

A quadruplet system based on four-letter codons rather than three has 256 total codons that could encode many more amino acids, independent of the natural triplet codon system. Expanding the number of protein building blocks would help to produce highly specialized proteins containing unnatural amino acids, potentially opening the door to advances in both basic biology and therapeutic applications (Hohsaka et al., 1996).

One approach to expanding the genetic code is based on a natural process called +1 frameshifting, where four rather than three nucleotide bases are effectively decoded as a single amino acid (Riyasaty and Atkins, 1968). The insertion of a single base generates a frameshift in an otherwise triplet codon gene, shifting the frame by one letter. So-called frameshift suppressor tRNAs allow protein synthesis to continue past this insertion to produce a normal full-length protein. For example, some tRNAs can read frameshift mutations, including insertions or deletions of one or two nucleotides in the mRNA. A tRNA that suppresses a +1 frameshift effectively reads or decodes a quadruplet codon (Figure 1).

**Figure 1.**
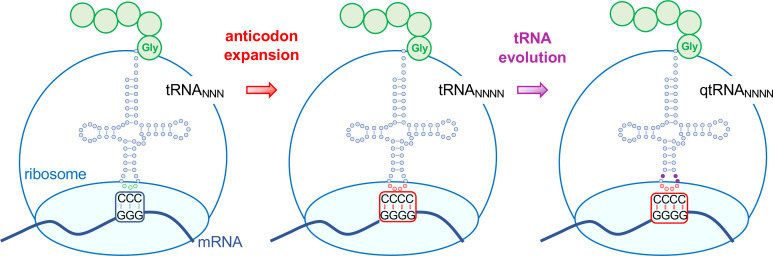
Evolving tRNAs to efficiently read quadruplet codons. The schematic illustrates the approach used by DeBenedictis et al. to evolve triplet-decoding tRNAs (left) into tRNAs with expanded anticodon loops (red dots) to decode quadruplet codons consisting of four nucleotide bases (N; middle), and ultimately into efficient quadruplet codon decoders (right). To test the efficiency of four-base translation, DeBenedictis et al. created reporter genes (such as luciferase) with a single base insertion (also referred to as +1 frameshift). The ability of a particular tRNA variant to read that +1-frameshift mutation as a four-base codon can be measured as a function of how much full length and active reporter protein (e.g., luciferase) is made in cells. DeBenedictis et al. then recorded the translation efficiency of tRNAs with simple mutations to expand the anticodon loop (middle, red dots). Next, tRNAs were evolved through various mutations into more effective quadruplet-decoding tRNAs (qtRNAs, purple dots, right). The work represents an important step towards engineering a quadruplet genetic code with 256 codons.

Image credit: Tarana Siddika, Ilka U. Heinemann, Patrick O’Donoghue (CC BY 4.0).

Translation using quadruplet codons exists in both natural and synthetic systems, so why did nature favor a triplet system overall? Now, in eLife, Erika DeBenedictis, Dieter Söll and Kevin Esvelt at the Massachusetts Institute of Technology and Yale University, report how tRNAs can be evolved to read quadruplet codons more efficiently (DeBenedictis et al., 2022). DeBenedictis et al. made simple mutations to several natural and synthetic tRNAs to generate various quadruplet-decoding RNAs (qtRNAs) based on the natural frameshifting variants and tested their efficiency in the bacterium *Escherichia coli*. This revealed that quadruplet codons only had a translation efficiency of 1%–3% compared to triplet codons. Some qtRNAs also showed significantly slowed growth rates in *E. coli*, while others were better tolerated despite their ability to translate quadruplet codons.

DeBenedictis et al. then tested whether tRNAs could evolve into qtRNAs through replacement of the entire anticodon (the complementary sequence to the codon located on the tRNA) with quadruplet codons using a phage-based library selection approach. This involved encoding modified qtRNAs into the genome of bacteriophages, which in turn infected the *E. coli* bacteria. Only functional qtRNAs enabled phages to successfully reproduce, thus highlighting effective quadruplet codon/anticodon pairs and comparing the efficiency of quadruplet decoding. Their data revealed that functional qtRNAS that can successfully decode quadruplet codons can arise through just a few mutations.

Next, the positions surrounding the anticodon were mutated across a large library of different qtRNAs (Figure 1). These experiments identified qtRNA variants that displayed up to 40-fold improvement in quadruplet decoding efficiency compared to unmutated quadruplet decoding tRNAs. Finally, mass spectrometry was used to show how some qtRNAs provided ‘high-fidelity’ four-base decoding and inserted just a single type of amino acid in response to a quadruplet codon. Other qtRNAs were less selective and the same four-base codon was ‘read’ with multiple different amino acids, revealing ambiguity as a potential limitation in quadruplet decoding.

Overall, the findings highlight the potential and limitations of a genetic code based on quadruplet codons. Quadruplet codons can be used to produce highly specialized proteins with new functionalities, but low translation fidelity and limited efficiency remain challenges in the field. DeBenedictis et al. use multiple approaches to mutagenize tRNAs, and they generated many improved qtRNA variants that represent a full palate of starting points to reassign quadruplet codons to new amino acids. The researchers demonstrated the power of tRNA variations on their own, and significant improvements in quadruplet decoding by engineering tRNAs alone. Combining qtRNAs with engineered tRNA synthetases to improve incorporation of unnatural amino acids (reviewed in Kim et al., 2022) or with genetically modified ribosomes to enhance 4-base translation (Dunkelmann et al., 2021) could further improve efficiency and decrease ambiguity in translating proteins with an increased codon size, thus vastly expanding the genetic code.

The work of DeBenedictis et al. points towards several areas worth further investigation to improve translation efficiency and fidelity with quadruplet codons and they show that in-roads can be made in both areas though tRNA engineering. Together with recent efforts to use quadruplet codons to encode synthetic amino acids (DeBenedictis et al., 2021; Dunkelmann et al., 2021), the current study suggests that the expansion of the genetic code with the help of quadruplet codons may be within reach.
